# Mountain Ultramarathon Induces Early Increases of Muscle Damage, Inflammation, and Risk for Acute Renal Injury

**DOI:** 10.3389/fphys.2018.01368

**Published:** 2018-10-08

**Authors:** Taisa Belli, Denise Vaz Macedo, Gustavo Gomes de Araújo, Ivan Gustavo Masselli dos Reis, Pedro Paulo Menezes Scariot, Fernanda Lorenzi Lazarim, Lázaro Alessandro Soares Nunes, René Brenzikofer, Claudio Alexandre Gobatto

**Affiliations:** ^1^Laboratory of Applied Sport Physiology (LAFAE), School of Applied Sciences, University of Campinas, Limeira, Brazil; ^2^Laboratory of Exercise Biochemistry (LABEX), Biochemistry Department, Biology Institute, University of Campinas, Campinas, Brazil; ^3^Laboratory of Instrumentation for Biomechanics (LIB), Faculty of Physical Education, University of Campinas, Campinas, Brazil

**Keywords:** muscle damage, inflammation, renal function, dehydration, ultramarathon

## Abstract

**Purpose:** This study aimed to investigate changes in muscle damage during the course of a 217-km mountain ultramarathon (MUM). In an integrative perspective, inflammatory response and renal function were also studied.

**Methods:** Six male ultra-runners were tested four times: pre-race, at 84 km, at 177 km, and immediately after the race. Blood samples were analyzed for serum muscle enzymes, acute-phase protein, cortisol, and renal function biomarkers.

**Results:** Serum creatine kinase (CK), lactate dehydrogenase (LDH), and aspartate aminotransferase (AST) increased significantly throughout the race (*P* < 0.001, *P* < 0.001; *P* = 0.002, respectively), and effect size (ES) denoted a large magnitude of muscle damage. These enzymes increased from pre-race (132 ± 18, 371 ± 66, and 28 ± 3 U/L, respectively) to 84 km (30, 1.8, and 3.9-fold, respectively); further increased from 84 to 177 km (4.6, 2.9, and 6.1-fold, respectively), followed by a stable phase until the finish line. Regarding the inflammatory response, significant differences were found for C-reactive protein (CRP) (*P* < 0.001) and cortisol (*P* < 0.001). CRP increased from pre-race (0.9 ± 0.3 mg/L) to 177 km (243-fold), cortisol increased from pre-race (257 ± 30 mmol/L) to the 84 km (2.9-fold), and both remained augmented until the finish line. Significant changes were observed for creatinine (*P* = 0.03), urea (*P* = 0.001), and glomerular filtration rate (GFR) (*P* < 0.001), and ES confirmed a moderate magnitude of changes in renal function biomarkers. Creatinine and urea increased, and GFR decreased from pre-race (1.00 ± 0.03 mg/dL, 33 ± 6 mg/dL, and 89 ± 5 ml/min/1.73 m^2^, respectively) to 84 km (1.3, 3.5, and 0.7-fold, respectively), followed by a plateau phase until the finish line.

**Conclusion:** This study shows evidence that muscle damage biomarkers presented early peak levels and they were followed by a plateau phase during the last segment of a 217-km MUM. The acute-phase response had a similar change of muscle damage. In addition, our data showed that our volunteers meet the risk criteria for acute kidney injury from 84 km until they finished the race, without demonstrating any clinical symptomatology.

## Introduction

Ultramarathons are foot race competitions comprising longer distances than a marathon and performed on a variety of terrains (Wu et al., 2004; Vernillo et al., 2016). Investigations have demonstrated different release patterns of muscle damage proteins during 130-km 2-day ultramarathon (Miyata et al., 2008; Arakawa et al., 2016), ∼500-, and 1,600-km continuous competitions (Davies et al., 1984; Fallon et al., 1999) compared to classical findings involving eccentric and prolonged exercises (Clarkson and Hubal, 2002; Brancaccio et al., 2010). However, it is difficult to generalize these findings in ultramarathons, given races range from 50 km through beyond 1,600 km, are performed on a mostly flat road or on varying terrains, and may occur in a single-stage or multi-days (Fallon et al., 1999; Wu et al., 2004; Mastaloudis et al., 2006; Miyata et al., 2008; Vernillo et al., 2016). Each of these characteristics may influence muscle damage during these competitions (Peake et al., 2005; Brancaccio et al., 2008; Banfi et al., 2012; Saugy et al., 2013). Thus, further studies are necessary to investigate this issue during ultramarathon races with different profiles. Pronounced muscle damage is often reported at the finish line of ∼200-km single-stage flat and mountain races (Nieman et al., 2005; Skenderi et al., 2006; Kim et al., 2007; Millet et al., 2011; Hoffman et al., 2012; Waskiewicz et al., 2012; Klapcinska et al., 2013; Son et al., 2015) with decreased response evidenced during recovery period (Millet et al., 2011; Klapcinska et al., 2013; Son et al., 2015). The kinetics of muscle damage biomarkers during the course of these competitions were also explored, with studies focused on races where most of the course is roughly flat (Kim et al., 2007; Waskiewicz et al., 2012; Klapcinska et al., 2013; Son et al., 2015). Here, we address this issue, and the focus and the originality of this study is to describe the kinetics of muscle damage and some related biomarkers during a ∼200-km single-stage mountain ultramarathon (MUM).

The etiology of exercise-induced muscle damage is related to the mechanical disruption of the fiber, disturbances in calcium homeostasis, and inflammatory processes (Clarkson and Sayers, 1999). Creatine kinase (CK), lactate dehydrogenase (LDH), and aspartate aminotransferase (AST) are among the most useful serum biomarkers of exercise-induced muscle damage. Generally, CK peaks about 12–24 h after downhill running, and it is markedly increased about 2–7 days after eccentric exercise (Clarkson and Hubal, 2002; Brancaccio et al., 2010). The time-to-peak for LDH and AST was 3 h after a half-marathon run, while serum CK activities were still increasing at 24 h after the competition (Lippi et al., 2008). Conversely, greater increases in CK were found during the second half of the 160–200 km flat races (Kim et al., 2007; Waskiewicz et al., 2012), with peak values of CK, LDH, and AST observed immediately after 166–280 km foot competitions (Millet et al., 2011; Klapcinska et al., 2013; Son et al., 2015). However, to our knowledge, no study purposed to investigate the kinetics of muscle damage during the course of a ∼200-km single-stage MUM. These competitions take place on off-road trails, coastal mountains, and paths with significant changes in elevation (Hoffman et al., 2012; Vernillo et al., 2016), which can result in greater muscle damage due the eccentric contractions (Peake et al., 2005; Banfi et al., 2012), and influence the muscle protein serum levels.

Reports of muscle soreness are also a common measure of muscle damage (Clarkson and Hubal, 2002). Classically, the intensity of discomfort initiates within the first 24 h post-exercise and peaks between 24 and 72 h (Cheung et al., 2003; Lewis et al., 2012). In addition, muscle damage initiates a series of immune reactions (Peake et al., 2005) and the production of a large number of acute-phase proteins (Pedersen and Hoffman-Goetz, 2000). C-reactive protein (CRP) is a classical acute-phase protein derived from a hepatocyte, which is fast synthesized and rises in the blood stream at high levels when the inflammatory responses is reaching its peak (Du Clos and Mold, 2004). Serum cortisol has an important immunomodulatory function in response to exercise (Nieman, 2000) and increases during prolonged competitions (Kupchak et al., 2014). Changes in serum CRP mimicked those found for CK during 160–200 km flat races (Kim et al., 2007; Waskiewicz et al., 2012), with no reports of the release pattern of CRP during MUM. Moreover, as far as we know, no studies investigated the kinetics of muscle soreness and serum cortisol during the course of these competitions.

Muscle damage rarely results in adverse consequences among athletes during ultramarathons (Hoffman et al., 2012). However, the release of excessive amounts of intramuscular proteins into the blood stream can negatively affect renal function, mainly in conditions of heat stress, dehydration, significant weight loss during the run, underlying renal problems, use of non-steroidal anti-inflammatory drugs during the race, high levels of competitiveness, and inadequate training (Hoffman et al., 2014; Lipman et al., 2017). Creatinine, urea, and glomerular filtration rate (GFR) are the major markers of renal function (Ricci et al., 2011), which have been substantially studied after ∼200-km foot races (Bruso et al., 2010; Hoffman et al., 2013; Hoffman and Weiss, 2016; Lipman et al., 2017), with some investigations focused on the kinetics of these biomarkers during the course of these competitions (Cairns and Hew-Butler, 2016; Lipman et al., 2014, 2016). Extending this understanding about kinetics of renal function markers can be clinically relevant for recognizing when medical attention is warranted during the course of these races.

Based on the above statements, this study aimed to investigate the changes in muscle damage during the course of a 217-km MUM. In an integrative perspective, inflammatory response and renal function were also analyzed during the race.

## Materials and Methods

### Participants

Six male experienced ultra-runners took part in the present study during their participation in the “Brazil 135 Ultramarathon.” Characteristics of athletes are presented on **Table 1**. Absence of metabolic syndrome, hypertension, and diabetes mellitus were confirmed by clinical history analysis, clinical examination, and laboratory tests. Volunteers had mild-to-moderate dyslipidemia (Catapano et al., 2016) and they reported no use of daily medication. Fifty-nine athletes participated in the solo race. Forty-two runners, including our volunteers, completed the competition and their race time ranged from 26 h and 43 min to 59 h and 43 min. This study was carried out in accordance with the recommendations of “Ethics Research Committee of the São Paulo State University (UNESP)” with written informed consent from all subjects. All subjects gave written informed consent in accordance with the Declaration of Helsinki. The protocol was approved by the “Ethics Research Committee of the São Paulo State University (UNESP)” (no. 037/2008).

**Table 1:** Demographic and training profile of athletes (*n* = 6)

Age (years)	47 ± 5
Height (cm)	170.7 ± 2.3
Body mass (kg)	73.1 ± 4.6
Body mass index [kg.(m^2^)^-1^]	24.9 ± 1.0
Waist circumference (cm)	88.6 ± 3.7
Fasting glycaemia (mg/dL)	73.5 ± 3.5
Triglyceride (mg/dL)	184.7 ± 44.8
Total cholesterol (mg/dL)	210.3 ± 13.0
High-density lipoprotein (mg/dL)	49.9 ± 7.5
Low-density lipoprotein (mg/dL)	136.7 ± 19.7
CHO/HDL	4.9 ± 1.0
LDL/HDL	3.4 ± 0.9
Running experience (years)	17 ± 5
Training volume (km/week)	118 ± 32
Ultramarathons raced (*n*)	7 ± 3
Marathons raced (*n*)	25 ± 10
Best marathon performance (km/h)	12.5 ± 0.9


*CHO/HDL, cholesterol/high-density lipoprotein ratio; LDL/HDL, low-density lipoprotein/high-density lipoprotein ratio.*

### Race Description and Research Design

**FIGURE 1:**
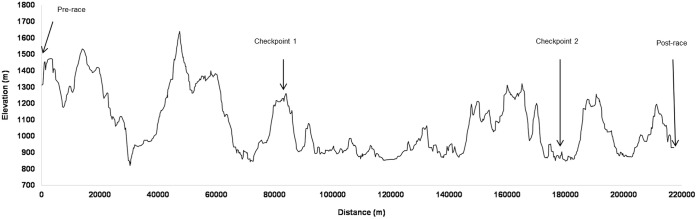
Elevation profile throughout the “Brazil 135 Ultramarathon” route with the four session test locations. Reprinted and adapted minimally from Belli et al. (2017b), Copyright (2017), with permission from Elsevier.

This field study occurred at the “Brazil 135 Ultramarathon.” This 217-km international foot race is a single-stage MUM performed on dirt roads in the most difficult segment of a Brazilian pilgrimage route called “The Walk of Faith” (Mantiqueira Mountains, Brazil), with a total positive and negative elevation change of 12,200 m (**Figure 1**). Runners may participate in the individual (solo) or relay race (teams comprising two, three, or four athletes) and must arrive at the finish line within 60 h to be considered an official finisher.

Volunteers were tested four times: pre-race (0 km), in two checkpoints during the race (84 and 177 km), and immediately after the race (217 km) for blood sampling, muscle soreness, and body mass (BM) measurements. Athletes also completed a questionnaire on basic demographics and pre-race training. Pre-race measurements were performed in the afternoon before the race at 5 h postprandial, whereas checkpoints and post-race evaluations occurred immediately after athletes reach these places. Volunteers could eat and drink *ad libitum* during the race. Ambient temperatures were 8–30°C.

### Blood Sampling and Analysis

Eight milliliters of blood samples were collected from each athlete by a certified nurse. Blood samples were collected into tubes with a coagulation enhancer and splitting gel (Vacuette, Greiner Bio-One^®^) and were immediately centrifuged (3000 rpm, 10 min). The blood serum was aliquoted and stored in liquid nitrogen. These samples were analyzed under laboratory conditions using an automatic device (Autolab 18, Boehringer Mannheim^®^) for CK, LDH, AST, creatinine, urea, total protein (Wiener kits^®^), and CRP (Biotécnica kit^®^). Fifty microliters of serum samples were added to polypropylene tubes with ^125^I (105 ml) in which it was decanted and had the radioactivity measured in gamma counter (1 min) for determination of cortisol concentration, in accordance to the commercial kit Coat-A-Count from Siemens^®^. All blood serum analysis results were corrected for changes in plasma volume estimated using the total protein (Ohira et al., 1977). GFR was estimated using the Cockroft-Gault equation (Cockcroft and Gault, 1976):

GFR=(140−age)×(body mass)/creatinine(mg/dL)×72

### Muscle Soreness

Runners rated their level of self-perceived muscle soreness by selecting a number that best described any general feeling of pain, soreness, and muscles ache using a 10-point Likert scale: 1 (no soreness), 2.5 (dull, vague ache), 4 (slight soreness), 5.5 (more than slight soreness), 7 (sore), 8.5 (very sore), and 10 (unbearably sore) (Nieman et al., 2005).

### Anthropometric Measurements

Body mass (kg) and height (cm) measurements were performed with volunteers barefooted and wearing light clothing, using a platform scale (Welmy^®^) with an accuracy of 0.1 kg and 0.5 cm, respectively. Body mass index [kg.(m^2^)^-1^] (BMI) was calculated as BM divided by the squared height.

### Race Performance

Race results were retrieved from the official race reports. To determine the speeds during sections of different distance and elevation, we calculated an equivalent flat speed as proposed by Saugy et al. (2013):

Equivalent flat speed=Equivalent flat distance(km)/race time(h)

Equivalent flat distance=distance(km)+(elevation change(m)/100)

### Statistical Analysis

Normal distribution and homogeneity of the data were verified by the Shapiro-Wilk and Levene’s tests, respectively. CK, LDH, and AST values were log transformed to reach a normal distribution before using parametric analysis. Repeated-measures ANOVAs were used for comparisons, and the Greenhouse-Geisser correction was applied if a violation of sphericity was pointed by Mauchly’s test. Scheffè *post hoc* test was performed when appropriate. Friedman’s ANOVA was used only for muscle soreness data. Then, Dunn’s *post hoc* test was performed when appropriate. Statistical significance was set at *P* < 0.05. Effect sizes (ESs) were calculated using partial eta squared (η^2^), which were classified as <0.2 (small); 0.2 to <0.8 (moderate); >0.8 (large). Data were expressed as mean ± standard error of mean (SEM). Statistical procedures were carried out using GraphPad Prism 6 (GraphPad Software, San Diego, CA, United States) and Statistic 7.0 (Statsoft, Tulsa, OK, United States).

## Results

**FIGURE 2:**
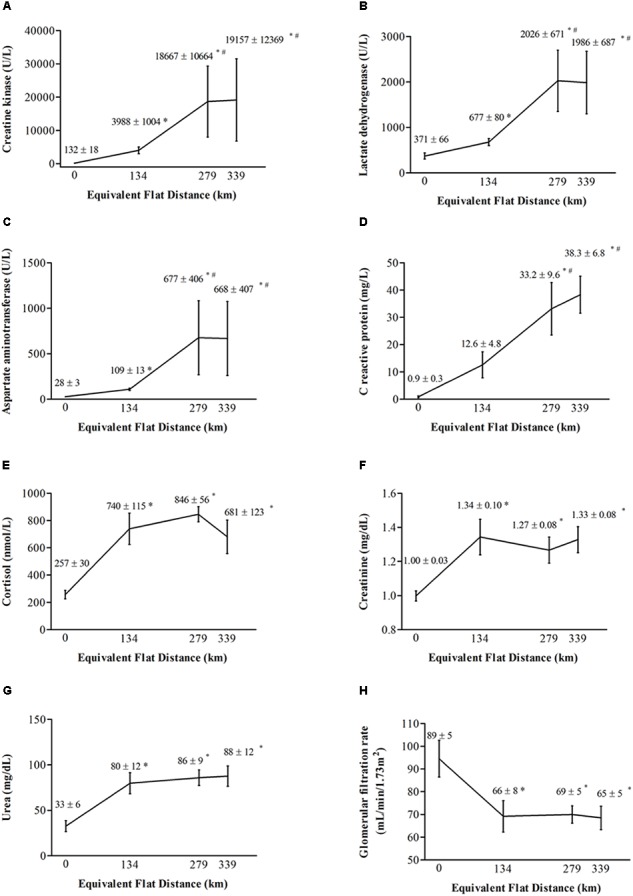
Serum concentrations of **(A)** creatine kinase, **(B)** lactate dehydrogenase, **(C)** aspartate aminotransferase, **(D)** C-reactive protein, **(E)** cortisol, **(F)** creatinine, **(G)** urea, and **(H)** glomerular filtration rate in athletes before the race, at the first and second checkpoints and immediately after the race, expressed as mean and standard error of mean. Race distance is expressed in equivalent flat distance. ^∗^Significant difference (*P* < 0.05) compared to before the race. ^#^Significant difference (*P* < 0.05) compared to the first checkpoint.

**Figure 2** illustrates muscle damage, inflammatory response, and renal function of ultra-runners throughout the race.

Significant increases were observed for CK [*F*_(3,15)_ = 105.55; *P* < 0.001; η^2^ = 0.95; large], LDH [*F*_(3,15)_ = 27.062; *P* < 0.001; η^2^ = 0.84; large], and AST [*F*_(1.09,5.48)_ = 26.977; *P* = 0.002; η^2^ = 0.84; large], denoting marked muscle damage during the competition. *Post hoc* analysis denoted serum CK, LDH, and AST increased from pre-race to the first checkpoint (*P* < 0.001; *P* = 0.04; *P* = 0.008, respectively), further increased from the first to the second checkpoint (*P* = 0.01; *P* = 0.006; *P* = 0.02, respectively), and were followed by a stable phase from the second checkpoint to post-race (*P* = 0.94; *P* = 0.99; *P* = 0.99, respectively).

Muscle soreness also changed significantly during the MUM (Friedman = 12.67347; *P* = 0.005). Although *post hoc* analysis denoted no changes from pre-race (1; no soreness) to the first checkpoint (3 ± 1; slight soreness) (*P* > 0.05), high reports of muscle soreness were observed from pre-race to the second checkpoint (7 ± 1; sore) (*P* < 0.05), followed by a stable phase from the second checkpoint to post-race (6 ± 1; sore) (*P* > 0.05).

Regarding inflammatory responses, significant differences were found for CRP [*F*_(3,15)_ = 14.899; *P* < 0.001; η^2^ = 0.75; moderate] and cortisol [*F*_(3,15)_ = 12.818; *P* < 0.001; η^2^ = 0.72; moderate] during the race. *Post hoc* analysis denoted that serum CRP increased significantly from pre-race (*P* = 0.002) and the first checkpoint (*P* = 0.04) to the second checkpoint, followed by a stable phase from the second checkpoint to post-race (*P* = 0.88). Serum cortisol increased significantly from pre-race to the first checkpoint (*P* = 0.002), followed by a stable phase from the first to the second checkpoint (*P* = 0.77) and from the second checkpoint to post-race (*P* = 0.47).

Significant changes were observed for creatinine [*F*_(3,15)_ = 7.0693; *P* = 0.03; η^2^ = 0.58; moderate], urea [*F*_(3,15)_ = 9.1867; *P* = 0.001; η^2^ = 0.65; moderate], and GFR [*F*_(3,15)_ = 11.127; *P* < 0.001; η^2^ = 0.69; moderate] during the competition. *Post hoc* analysis denoted creatinine and urea increased, and GFR decreased significantly from pre-race to the first checkpoint (*P* = 0.01; *P* = 0.01; *P* = 0.003, respectively), followed by a stable phase from the first to the second checkpoint (*P* = 0.87; *P* = 0.96; *P* = 0.99, respectively) and from the second checkpoint to post-race (*P* = 0.92; *P* = 0.99; *P* = 0.99, respectively).

Body mass also changed significantly during the race [*F*_(3,15)_ = 15.155; *P* < 0.001; η^2^ = 0.75; moderate]. *Post hoc* analysis showed significant decreases in BM values observed from pre-race (73.1 ± 4.6 kg) to the first checkpoint (70.4 ± 4.7 kg, *P* = 0.006), followed by a stable phase from the first to the second checkpoint (69.0 ± 4.5 kg, *P* = 0.24) and from the second checkpoint to post-race (70.0 ± 4.4 kg; *P* = 0.44).

The race time of our volunteers was 53.8 ± 3.1 h, their final rank being from 10th to 38th position out of 42 finishers. **Table 2** presents the equivalent flat speed and race time of ultra-runners during the competition. Equivalent flat speed changed significantly during the race [*F*_(2,10)_= 14.159; *P* = 0.001; η^2^ = 0.74; moderate]. *Post hoc* analysis denoted athletes ran at higher speeds in the first segment (distance = 0–84 km) compared to the second (distance = 84–177 km) (*P* = 0.001) and third segments of the race (distance = 177–217 km) (*P* = 0.02). The race time also changed significantly during the race [*F*_(2.10)_ = 82.101; *P* < 0.001; η^2^ = 0.94; large]. Race time was longer during the second segment compared to the first (*P* < 0.001) and third segments of the race (*P* < 0.001).

**Table 2:** Ultramarathon race performance measure.

	0–84 km	84–177 km	177–217 km
Equivalent flat speed (km/h)	8.5 ± 0.9	5.4 ± 0.3^∗^	6.4 ± 0.7^∗^
Race time (h)	16.5 ± 1.4	27.2 ± 1.5^∗^	9.9 ± 0.9^∗,#^
Equivalent flat distance (km)	134	145	60
Distance (km)	84	93	40
Elevation change (m)	5046.4	5114.4	2039.1


*^∗^*P* < 0.05 compared to 0–84 km; ^#^*P* < 0.05 compared to 84–177 km.*

## Discussion

In this investigation, we explored changes in muscle damage during the course of a 217-km MUM. We also studied the inflammatory response and renal function in our athletes during the race. Our main findings indicated early peak values of muscle damage biomarkers (43.8 ± 2.5 h of the race) and a plateau phase during the last segment of the race (from 43.8 ± 2.5 to 53.8 ± 3.1 h). The acute-phase response had a similar change of muscle damage, whereas cortisol and renal biomarkers had greater changes from the 16.5 ± 1.4 h into the race and maintained it until the finish line of the competition.

The time and amount of extracellular release and clearance from the plasma of muscle proteins basically depends on the individual training level, the intensity and duration of exercise, and biochemical characteristics of the molecule (Bessa et al., 2008; Brancaccio et al., 2008), and suffers high individual biological variation (Nunes et al., 2010). Muscle damage responses found herein were assessed in middle-aged amateur ultra-runners, who had long running experience, performed a long distance of running volume per week, and had their final rank from the 10th place during the competition. The highest exercise intensity of athletes during the first segment of the race may have influenced the initial increases in muscle damage found at the first checkpoint. In addition, the longest race time during the second segment may be related to additional increases in muscle damage observed at the second checkpoint of the competition. Then, a lower susceptibility of skeletal muscle to damage following repeated bouts of the same exercise (Peake et al., 2005) and an equilibrium between the rate of loss of proteins from cells and the rate of clearance of proteins from the blood (Son et al., 2015) may be mechanisms underlying the plateau in serum biomarkers during the last segment of the race. An interesting finding of this study was the similar response of CK (∼43-45 kDa), LDH (∼140 kDa), and AST (∼90 kDa), despite their different molecular size, which commonly influences these responses during exercise (Bessa et al., 2008; Brancaccio et al., 2010). High individual variation of serum levels of muscle proteins observed herein is well documented and is commonly related to age, race, and muscle mass (Brancaccio et al., 2008). Moreover, we reported recently that *ACTN3 R577X* gene polymorphism may also influence the magnitude of muscle damage during an ultra-endurance race (Belli et al., 2017a).

Our findings expand on a limited number of previous studies showing an earlier increase of muscle damage biomarkers during ∼200-km single-stage ultramarathon competitions (Kim et al., 2007; Waskiewicz et al., 2012; Kupchak et al., 2014; Son et al., 2015) than classically described (Clarkson and Hubal, 2002; Lippi et al., 2008; Brancaccio et al., 2010). Kim et al. (2007) concur reporting that 54 male ultra-runners (45.7 ± 5.1 years old) had higher increases in serum CK (90-fold from the pre-race value) during the second half of a 200-km competition (race time: 23 h and 53 min to 34 h and 56 min) in Cheju Island (at sea level), South Korea. Corroborating these results, Son et al. (2015) found greater increases in serum CK at 150 km (71-fold) and 200-km (98-fold) in 32 male ultra-distance runners (56–70 years old) during the same race (race time: 29 h and 18 min to 33 h and 59 min) in South Korea. Waskiewicz et al. (2012) also observed that 14 male amateur runners (43.0 ± 10.8 years old) had greater increases in serum CK (70-fold from pre-race values) between 94.6 km (12 h) and 168.5 km (24 h) during a 24-h flat race. In addition, consistent with our findings, Klapcinska et al. (2013) documented that seven male amateur athletes (45.4 ± 9.2 years old) had greater increases in serum CK (93-fold from pre-race values) ∼170 km of race (24 h), followed by a stable phase (106-fold from pre-race values) up to ∼280 km (48 h) during a 48-h ultramarathon run performed in a perfectly flat area in Katowice, Poland.

Besides the similar kinetics, an interesting finding of this investigation was the comparable serum CK in the study volunteers than reported in athletes during the course of foot races of similar distance but on flat terrain. Mean values of 4284–5056, 17,502-18,010, and 20,605 U/L were observed at 94–102, ∼170, and ∼280 km of single-stages flat races, respectively (Waskiewicz et al., 2012; Klapcinska et al., 2013), which are very close by the mean values of 3988, 18667, and 19157 U/L at 84 km, 177 km, and at finished line observed herein during a single-stage 217 km MUM. However, the study athletes covered these respective distances with 5046, 10,161, and 12,200 m of cumulative elevation changes, which could trigger greater increases in muscle damage. In line with this, downhill running involves a large eccentric component. As the study athletes stride down the coastal mountains, the contracting quadriceps muscle controls the rate of knee flexion against the force of gravity, and the muscle undergoes an eccentric contraction with each stride (Proske and Morgan, 2001). Eccentric contractions cause more damage than concentric contractions because fewer motor units are recruited during eccentric exercise, and therefore, a reduced cross-sectional area is activated to handle the same load as would be handled in a concentric exercise (Clarkson and Sayers, 1999). Moreover, during eccentric exercise the contracting muscle is forcibly lengthened, and the extent of muscle damage is related to the change in the muscle length (Proske and Morgan, 2001). Slower pace provide a possible explanation for the similar CK values observed herein than during flat races. The study athletes covered the reported distances at higher race times (16.5, 43.8, and 53.8 h, respectively) than aforementioned races (12, 24, and 48 h, respectively). This is in agreement with lower CK values (3719 U/L) observed after a 330-km MUM despite the extremely high downhill, probably as a result of the very low concentric/eccentric contraction intensity due to the slow pace of the athletes (Saugy et al., 2013), and expands the knowledge that the amount of muscle damage is not necessarily related to the elevation even during the course of a MUM.

The highest increases in serum CRP coincided with the highest levels of serum muscle proteins and reports of muscle soreness in our volunteers, followed by a stable phase in all of these biomarkers. It denotes that the inflammatory response occurred in parallel to muscle damage during the course of the race. Corroborating this finding, Kim et al. (2007) and Waskiewicz et al. (2012) observed that increases in serum CRP concurred to the increases in serum CK throughout 160–200 km flat races. The greatest concentrations in serum cortisol occurred earlier, from the first checkpoint of the race, and these values remained augmented until the finish line in our athletes. In addition to the immunosuppressive effect of cortisol, it serves as a mechanism to increase blood glucose levels, and to assist in fat and protein metabolism (Kupchak et al., 2014), which may explain this earlier increase in serum cortisol compared to increases in serum CRP during a 217-km MUM. This result expands previous reports of higher levels of cortisol after 160-km foot races (Kraemer et al., 2008; Kupchak et al., 2014).

The increases in serum creatinine in the study athletes were 1.3 ± 0.1, 1.3 ± 0.1, and 1.3 ± 0.1-fold from pre-race values to the first checkpoint, the second checkpoint, and post-race evaluations; and the percent decrease in GFR from the starting values seen in our volunteers were -27 ± 4, -25 ± 5, and -26 ± 5% for each moment of evaluation. According to Risk, Injury, Failure, Loss of function, and End-stage renal disease (RIFLE) classification (Ricci et al., 2011), the increases in serum creatinine observed herein did not meet the risk criteria for acute kidney injury (i.e., increased serum creatinine × 1.5), whereas decreases in GFR meet this criteria (i.e., >25% decrease in GFR). However, none of the ultra-runners in the study experienced an adverse medical event requiring medical attention during or after the race. Our findings expand on a limited number of prior studies showing an early increase of risk criteria for acute renal injury during ∼200-km foot races. Cairns and Hew-Butler (2016) concurred, reporting decreases in GFR greater than 25% from baseline values in 10 athletes (40.7 ± 10.3 to 50.6 ± 11.0 years old) from the 53-km during a 100-to 170-km MUM in Australia (race time: 24.6 ± 9.8 h to 26.5 ± 8.3 h), without demonstrating clinical symptomatology. Lipman et al. (2014) also observed decreases in GFR greater than 25% from pre-race values in 30 participants (39.6 ± 10.6 years old) from the 40-km during 250-km multi-stage races performed in Gobi Desert, China; Sahara desert, Egypt; and Namibian desert.

The muscle breakdown products from rhabdomyolysis cause renal arteriolar vasoconstriction and contribute to increase the risk of renal dysfunction (Holt and Moore, 2001). However, we found that further increases in CK, LDH, and AST during the second checkpoint of the race (177 km) did not occur in parallel with additional decreases in renal function. The present findings are consistent with previous investigation in a 160-km MUM (Bruso et al., 2010), which suggest that although CK levels have been shown to be a predictor of clinical course in non-exercising scenarios, it may not be especially useful in distinguishing those susceptible to renal dysfunction during these races. Dyslipidemia can take part of the development and progression of renal disease in non-exercising conditions (i.e. lipid nephrotoxicity) (Gyebi et al., 2012) through inflammatory stress, oxidative stress, endoplasmic reticulum stress, endothelial dysfunction, and activation of the renin–angiotensin system (Ruan et al., 2009). However, despite the study volunteers had mild-to-moderate dyslipidemia, which may have influenced increases in risk for acute renal injury found during the competition, the hypothesis of lipid-mediated renal injury in exercising scenarios remains to be clarified. Dehydration may contribute to decrease renal perfusion, compounded the risk for acute kidney injury during an ultramarathon (Lipman et al., 2017). Changes in BM provide a simple and accurate index to estimate hydration status during exercise (Armstrong, 2007). When used for monitor dehydration, some BM reduction is awaited and seems to be well tolerated during ultramarathons (Hoffman et al., 2014). Given the combination of substrate utilization and the liberation of glycogen-bound water during exercise, euhydration requires BM decrease of at least 2–5% during these competitions (Hoffman et al., 2018a). Here, the decreases in BM from pre-race values were -3.8 ± 0.7, -5.6 ± 1.4, and -4.1 ± 0.7% for each point of evaluation, with three athletes presented reductions in BM higher than 5% (-5.2 to -10.4%) and concomitant decreases in GRF higher than 25% (-27.1 to -44.4%) from the first checkpoint (84 km) of the race. The present finding show evidences of a negative effect of dehydration on renal function since early stages of a ∼200-km single stage MUM. Greater BM loss were also associated with renal impairment in ultra-runners (*n* = 128; 39.6 ± 8.9 years old) from the 120-km during 250-km multi-stage races performed in Jordanian desert, Jordan; the Atacama Desert, Chile; and Gobi Desert, China (Lipman et al., 2016).

Recent literature were the first to propose medical consensus guidelines (Hoffman et al., 2014, 2015), nutrition recommendation (Costa et al., 2018), and hydration guidance (Hoffman et al., 2018a,b) applied to ultra-endurance races. Medical guidelines elucidate medical care to the most common serious problems that may affect athletes during these events, including exertional rhabdomyolysis and related development of acute kidney injury (Hoffman et al., 2014). Most single-stage MUM, as Brazil 135 Ultramarathon, are located in remote areas, where access may be extremely limited. It constitutes a challenge to organization of a medical support system for these events (Hoffman et al., 2014, 2015). Therefore, recognizing some segments during the course of these races when medical attention is required may constitute a valuable background to deal with this challenge. Here, we addressed this issue and present novel insights about the kinetics of renal biomarkers during a ∼200-km MUM. Our data showed evidences that the study volunteers meet the risk criteria for acute kidney injury from the early segments (∼16 h into the race) until finish line of the race, which was possibly associated with dehydration, and without they demonstrated any clinical symptomatology. Moreover, each ultra-endurance event will have some unique issues in terms of medical needs, which depends on a variety of specific characteristics of the competition (Hoffman et al., 2014). In line with this, the present investigation is the first one to elucidate a number of physiological responses during the “Brazil 135 Ultramarathon,” and the data presented here may constitute an important knowledge to pre-race planning of medical services applied to next editions of this event.

### Limitations

We acknowledge some limitations to this study. The number of the subjects is assumed as a limitation. Although further research is required in larger population groups, the findings presented herein are representative of a wide range of ultra-runners who are able to finish the MUM within the mandatory race time, including most of the people who perform in these competitions. We also highlight the difficult to access this sample during the course of a single-stage MUM, which subjects must conclude the race within a mandatory finish time. Another limitation is related to collect balanced data sets. The two first segments evaluated during the race were balanced in terms of distance and elevation change, whereas the third segment comprised a shorter course with proportional elevation change than former segments. However, to our knowledge, the observational design of this study (i.e., athletes evaluated in two checkpoints during a MUM) is unique and the first one allowing the investigation of the kinetics of muscle damage and related biomarkers during a ∼200-km single-stage MUM, in spite of unfavorable and challenging environment at field research (i.e., dirt roads on coastal mountain). Thus, although unbalanced field collection may appear, it is important to emphasize that exertional rhabdomyolysis and related development of acute kidney injury are serious medical problems that may affect athletes during these events (Hoffman et al., 2014) and the lessons learned here are far beyond what we can observe within well-controlled laboratories conditions, as previously highlighted in a field study about hyponatremia in ultramarathon (Cairns and Hew-Butler, 2016). Finally, we also assumed only conventional clinical tests were employed to evaluate the study athletes, and this is both a limitation and an advantage. The measurements of some novel markers would be interesting to better explore the study responses during the course of the race, whereas conventional clinical test are usually more accessible, with the present findings being able to be more widely used as reference in practical situations to monitor the athletes.

### Future Directions

Future investigations may focus on evaluating the study responses in top finishers during the course of a MUM, since it is well documented that muscle damage is associated with exercise intensity and duration (Banfi et al., 2012) and individual training level (Brancaccio et al., 2008). Future studies may be able to design more balanced segments to the evaluation of the ultra-runners throughout a MUM, and thus better explore the plateau phase in biomarkers observed in this study during the last course of the race. Finally, in addition to early increases observed in this study, investigations denoted muscle damage and inflammation biomarkers were decreased 1–2 days after 200–280 km flat races (Klapcinska et al., 2013; Son et al., 2015) and returned to pre-race levels 9 days after a 166-km MUM (Millet et al., 2011). Prior studies also denoted a full recovery of resting renal biomarkers after 40- to 80-km stages of a 250-km multi-stage race (Lipman et al., 2014), with no cumulative effect found after subsequent editions of a 160-MUM (Hoffman and Weiss, 2016). On the other hand, some investigations evidenced alterations of resting renal biomarkers persisting during the recovery period after 90- to 100-km foot races (Irving et al., 1990; Kao et al., 2015), which evidences the importance of future studies to clarify this issue during ultramarathons of different profiles.

## Conclusion

This study shows, in a relatively small group of ultra-runners taking part in a 217-km MUM, evidence that muscle damage biomarkers presented earlier peak levels (∼44 h into the race) than classically reported during eccentric and prolonged exercises (12 h to 7 days after the exercise) (Brancaccio et al., 2010), and they were followed by a plateau phase during the last segment (∼44 to 54 h into the race) of a 217-km MUM. The acute-phase protein showed a similar response of muscle damage biomarkers. These findings are novel, and expand on a limited number of previous investigations conducted during flat ultramarathons. In addition, our data also showed that the study volunteers meet the risk criteria for acute kidney injury from the first checkpoint (∼16 h into the race) until they finished the race, without demonstrating any clinical symptomatology.

## Author Contributions

TB and CG designed the study, conducted analyses, and wrote the manuscript. PS, GdA, IdR, FL, and LN assisted in acquisition, analysis, and interpretation of data, and reviewed and edited the article. DM and RB made substantial contribution including conception and design of the study, and a critical revision of the article. All authors read and approved the final manuscript.

## Conflict of Interest Statement

The authors declare that the research was conducted in the absence of any commercial or financial relationships that could be construed as a potential conflict of interest.

## References

[B1] Arakawa K. Hosono A. Shibata K. Ghadimi R. Fuku M. Goto C. (2016). Changes in blood biochemical markers before, during, and after a 2-day ultramarathon. *Open Access J. Sports Med.* 7 43–50. 10.2147/OAJSM.S97468 27186145 PMC4847591

[B2] Armstrong L. E. (2007). Assessing hydration status: the elusive gold standard. *J. Am. Coll. Nutr.* 26 575S–584S. 10.1080/07315724.2007.10719661 17921468

[B3] Banfi G. Colombini A. Lombardi G. Lubkowska A. (2012). Metabolic markers in sports medicine. *Adv. Clin. Chem.* 56 1–54. 10.1016/B978-0-12-394317-0.00015-7 22397027

[B4] Belli T. Crisp A. H. Verlengia R. (2017a). Greater muscle damage in athletes with ACTN3 R577X (rS1815739) gene polymorphism after an ultra-endurance race: a pilot study. *Biol. Sport* 34 105–110. 10.5114/biolsport.2017.64583 28566803 PMC5424449

[B5] Belli T. de Macedo D. M. Scariot P. P. M. de Araújo G. G. dos Reis I. G. M. Lazarim F. L. (2017b). Glycemic control and muscle damage in 3 athletes with type 1 diabetes during a successful performance in a relay ultramarathon: a case report. *Wilderness Environ. Med.* 28 239–245. 10.1016/j.wem.2017.04.005 28629959

[B6] Bessa A. Nissenbaum M. Monteiro A. Gandra P. G. Nunes L. S. Bassini-Cameron A. (2008). High-intensity ultraendurance promotes early release of muscle injury markers. *Br. J. Sports Med.* 42 889–893. 10.1136/bjsm.2007.043786 18203867

[B7] Brancaccio P. Lippi G. Maffulli N. (2010). Biochemical markers of muscular damage. *Clin. Chem. Lab. Med.* 48 757–767. 10.1515/CCLM.2010.179 20518645

[B8] Brancaccio P. Maffulli N. Buonauro R. Limongelli F. M. (2008). Serum enzyme monitoring in sports medicine. *Clin. Sports Med.* 27 1–18. 10.1016/j.csm.2007.09.005 18206566

[B9] Bruso J. R. Hoffman M. D. Rogers I. R. Lee L. Towle G. Hew-Butler T. (2010). Rhabdomyolysis and hyponatremia: a cluster of five cases at the 161-km 2009 Western states endurance run. *Wilderness Environ. Med.* 21 303–308. 10.1016/j.wem.2010.06.012 21168782

[B10] Cairns R. S. Hew-Butler T. (2016). Proof of concept: hypovolemic hyponatremia may precede and augment creatine kinase elevations during an ultramarathon. *Eur. J. Appl. Physiol.* 116 647–655. 10.1007/s00421-015-3324-4 26747653

[B11] Catapano A. L. Graham I. De Backer G. Wiklund O. Chapman M. J. Drexel H. (2016). 2016 ESC/EAS guidelines for the management of dyslipidaemias. *Eur. Heart J.* 37 2999–3058. 10.1093/eurheartj/ehw272 27567407

[B12] Cheung K. Hume P. Maxwell L. (2003). Delayed onset muscle soreness : treatment strategies and performance factors. *Sports Med.* 33 145–164. 10.2165/00007256-200333020-00005 12617692

[B13] Clarkson P. M. Hubal M. J. (2002). Exercise-induced muscle damage in humans. *Am. J. Phys. Med. Rehabil.* 81 S52–S69. 10.1097/01.PHM.0000029772.45258.43 12409811

[B14] Clarkson P. M. Sayers S. P. (1999). Etiology of exercise-induced muscle damage. *Can. J. Appl. Physiol.* 24 234–248. 10.1139/h99-020 10364418

[B15] Cockcroft D. W. Gault M. H. (1976). Prediction of creatinine clearance from serum creatinine. *Nephron* 16 31–41. 10.1159/000180580 1244564

[B16] Costa R. J. S. Hoffman M. D. Stellingwerff T. (2018). Considerations for ultra-endurance activities: part 1- nutrition. *Res. Sports Med.* 28 1–16. 10.1080/15438627.2018.1502188 30056753

[B17] Davies B. Shapiro C. M. Daggett A. Gatt J. A. Jakeman P. (1984). Physiological changes and sleep responses during and following a world record continuous walking record. *Br. J. Sports Med.* 18 173–180. 10.1136/bjsm.18.3.173 6487943 PMC1859379

[B18] Du Clos T. W. Mold C. (2004). C-reactive protein: an activator of innate immunity and a modulator of adaptive immunity. *Immunol. Res.* 30 261–277. 10.1385/IR:30:3:261 15531769

[B19] Fallon K. E. Sivyer G. Sivyer K. Dare A. (1999). The biochemistry of runners in a 1600 km ultramarathon. *Br. J. Sports Med.* 33 264–269. 10.1136/bjsm.33.4.264 10450482 PMC1756186

[B20] Gyebi L. Soltani Z. Reisin E. (2012). Lipid nephrotoxicity: new concept for an old disease. *Curr. Hypertens. Rep.* 14 177–181. 10.1007/s11906-012-0250-2 22290079

[B21] Hoffman M. D. Goulet E. D. B. Maughan R. J. (2018a). Considerations in the use of body mass change to estimate change in hydration status during a 161-kilometer ultramarathon running competition. *Sports Med.* 48 243–250. 10.1007/s40279-017-0782-3 28895063

[B22] Hoffman M. D. Stellingwerff T. Costa R. J. S. (2018b). Considerations for ultra-endurance activities: part 2 - hydration. *Res. Sports Med.* 28 1–13. 10.1080/15438627.2018.1502189 30056755

[B23] Hoffman M. D. Ingwerson J. L. Rogers I. R. Hew-Butler T. Stuempfle K. J. (2012). Increasing creatine kinase concentrations at the 161-km Western states endurance run. *Wilderness Environ. Med.* 23 56–60. 10.1016/j.wem.2011.11.001 22441091

[B24] Hoffman M. D. Pasternak A. Rogers I. R. Khodaee M. Hill J. C. Townes D. A. (2014). Medical services at ultra-endurance foot races in remote environments: medical issues and consensus guidelines. *Sports Med.* 44 1055–1069. 10.1007/s40279-014-0189-3 24748459

[B25] Hoffman M. D. Rogers I. R. Joslin J. Asplund C. A. Roberts W. O. Levine B. D. (2015). Managing collapsed or seriously ill participants of ultra-endurance events in remote environments. *Sports Med.* 45 201–212. 10.1007/s40279-014-0270-y 25326844

[B26] Hoffman M. D. Stuempfle K. J. Fogard K. Hew-Butler T. Winger J. Weiss R. H. (2013). Urine dipstick analysis for identification of runners susceptible to acute kidney injury following an ultramarathon. *J. Sports Sci.* 31 20–31. 10.1080/02640414.2012.720705 23035796

[B27] Hoffman M. D. Weiss R. H. (2016). Does Acute Kidney Injury From an Ultramarathon Increase the Risk for Greater Subsequent Injury? *Clin. J. Sport Med.* 26 417–422. 10.1097/JSM.0000000000000277 26657822 PMC4900946

[B28] Holt S. G. Moore K. P. (2001). Pathogenesis and treatment of renal dysfunction in rhabdomyolysis. *Intensive Care Med.* 27 803–811. 10.1007/s001340100878 11430535

[B29] Irving R. A. Noakes T. D. Raine R. I. Van Zyl Smit R. (1990). Transient oliguria with renal tubular dysfunction after a 90 km running race. *Med. Sci. Sports Exerc.* 22 756–761. 10.1249/00005768-199012000-00004 2287252

[B30] Kao W. F. Hou S. K. Chiu Y. H. Chou S. L. Kuo F. C. Wang S. H. (2015). Effects of 100-km ultramarathon on acute kidney injury. *Clin. J. Sport. Med.* 25 49–54. 10.1097/JSM.0000000000000116 24949829

[B31] Kim H. J. Lee Y. H. Kim C. K. (2007). Biomarkers of muscle and cartilage damage and inflammation during a 200 km run. *Eur. J. Appl. Physiol.* 99 443–447. 10.1007/s00421-006-0362-y 17206443

[B32] Klapcinska B. Waskiewicz Z. Chrapusta S. J. Sadowska-Krepa E. Czuba M. Langfort J. (2013). Metabolic responses to a 48-h ultra-marathon run in middle-aged male amateur runners. *Eur. J. Appl. Physiol.* 113 2781–2793. 10.1007/s00421-013-2714-8 24002469 PMC3824198

[B33] Kraemer W. J. Fragala M. S. Watson G. Volek J. S. Rubin M. R. French D. N. (2008). Hormonal responses to a 160-km race across frozen Alaska. *Br. J. Sports Med.* 42 116–120; discussion120. 10.1136/bjsm.2007.035535 17638844

[B34] Kupchak B. R. Kraemer W. J. Hoffman M. D. Phinney S. D. Volek J. S. (2014). The impact of an ultramarathon on hormonal and biochemical parameters in men. *Wilderness Environ. Med.* 25 278–288. 10.1016/j.wem.2014.03.013 24931590

[B35] Lewis P. B. Ruby D. Bush-Joseph C. A. (2012). Muscle soreness and delayed-onset muscle soreness. *Clin. Sports Med.* 31 255–262. 10.1016/j.csm.2011.09.009 22341015

[B36] Lipman G. S. Krabak B. J. Rundell S. D. Shea K. M. Badowski N. Little C. (2016). Incidence and prevalence of acute kidney injury during multistage ultramarathons. *Clin. J. Sport. Med.* 26 314–319. 10.1097/JSM.0000000000000253 26513390

[B37] Lipman G. S. Krabak B. J. Waite B. L. Logan S. B. Menon A. Chan G. K. (2014). A prospective cohort study of acute kidney injury in multi-stage ultramarathon runners: the Biochemistry in endurance runner study (BIERS). *Res. Sports Med.* 22 185–192. 10.1080/15438627.2014.881824 24650338

[B38] Lipman G. S. Shea K. Christensen M. Phillips C. Burns P. Higbee R. (2017). Ibuprofen versus placebo effect on acute kidney injury in ultramarathons: a randomised controlled trial. *Emerg. Med. J.* 34 637–642. 10.1136/emermed-2016-206353 28679502

[B39] Lippi G. Schena F. Salvagno G. L. Montagnana M. Gelati M. Tarperi C. (2008). Acute variation of biochemical markers of muscle damage following a 21-km, half-marathon run. *Scand. J. Clin. Lab. Invest.* 68 667–672. 10.1080/00365510802126844 18609111

[B40] Mastaloudis A. Traber M. G. Carstensen K. Widrick J. J. (2006). Antioxidants did not prevent muscle damage in response to an ultramarathon run. *Med. Sci. Sports Exerc.* 38 72–80. 10.1249/01.mss.0000188579.36272.f6 16394956

[B41] Millet G. Y. Tomazin K. Verges S. Vincent C. Bonnefoy R. Boisson R. C. (2011). Neuromuscular consequences of an extreme mountain ultra-marathon. *PLoS One* 6:e17059. 10.1371/journal.pone.0017059 21364944 PMC3043077

[B42] Miyata M. Kasai H. Kawai K. Yamada N. Tokudome M. Ichikawa H. (2008). Changes of urinary 8-hydroxydeoxyguanosine levels during a two-day ultramarathon race period in Japanese non-professional runners. *Int. J. Sports Med.* 29 27–33. 10.1055/s-2007-965072 17614024

[B43] Nieman D. C. (2000). Special feature for the olympics: effects of exercise on the immune system: exercise effects on systemic immunity. *Immunol. Cell Biol.* 78 496–501. 10.1111/j.1440-1711.2000.t01-5-.x 11050532

[B44] Nieman D. C. Dumke C. L. Henson D. A. McAnulty S. R. Gross S. J. Lind R. H. (2005). Muscle damage is linked to cytokine changes following a 160-km race. *Brain Behav. Immun.* 19 398–403. 10.1016/j.bbi.2005.03.008 16061149

[B45] Nunes L. A. Brenzikofer R. de Macedo D. V. (2010). Reference change values of blood analytes from physically active subjects. *Eur. J. Appl. Physiol.* 110 191–198. 10.1007/s00421-010-1493-8 20446091

[B46] Ohira Y. Ito A. Ikawa S. (1977). Correction of water content and solute concentration in blood during hemoconcentration. *J. Appl. Physiol. Respir. Environ. Exerc. Physiol.* 42 739–743. 863843 10.1152/jappl.1977.42.5.739

[B47] Peake J. Nosaka K. Suzuki K. (2005). Characterization of inflammatory responses to eccentric exercise in humans. *Exerc. Immunol. Rev.* 11 64–85. 16385845

[B48] Pedersen B. K. Hoffman-Goetz L. (2000). Exercise and the immune system: regulation, integration, and adaptation. *Physiol. Rev.* 80 1055–1081. 10.1152/physrev.2000.80.3.1055 10893431

[B49] Proske U. Morgan D. L. (2001). Muscle damage from eccentric exercise: mechanism, mechanical signs, adaptation and clinical applications. *J. Physiol.* 537 333–345. 10.1111/j.1469-7793.2001.00333.x 11731568 PMC2278966

[B50] Ricci Z. Cruz D. N. Ronco C. (2011). Classification and staging of acute kidney injury: beyond the RIFLE and AKIN criteria. *Nat. Rev. Nephrol.* 7 201–208. 10.1038/nrneph.2011.14 21364520

[B51] Ruan X. Z. Varghese Z. Moorhead J. F. (2009). An update on the lipid nephrotoxicity hypothesis. *Nat. Rev. Nephrol.* 5 713–721. 10.1038/nrneph.2009.184 19859071

[B52] Saugy J. Place N. Millet G. Y. Degache F. Schena F. Millet G. P. (2013). Alterations of neuromuscular function after the world’s most challenging mountain ultra-marathon. *PLoS One* 8:e65596. 10.1371/journal.pone.0065596 23840345 PMC3694082

[B53] Skenderi K. P. Kavouras S. A. Anastasiou C. A. Yiannakouris N. Matalas A. L. (2006). Exertional Rhabdomyolysis during a 246-km continuous running race. *Med. Sci. Sports Exerc.* 38 1054–1057. 10.1249/01.mss.0000222831.35897.5f 16775544

[B54] Son H. J. Lee Y. H. Chae J. H. Kim C. K. (2015). Creatine kinase isoenzyme activity during and after an ultra-distance (200 km) run. *Biol. Sport* 32 357–361. 10.5604/20831862.1163384 28479667 PMC5394848

[B55] Vernillo G. Savoldelli A. Skafidas S. Zignoli A. La Torre A. Pellegrini B. (2016). An extreme mountain ultra-marathon decreases the cost of uphill walking and running. *Front. Physiol.* 7:530. 10.3389/fphys.2016.00530 27877137 PMC5100553

[B56] Waskiewicz Z. Klapcinska B. Sadowska-Krepa E. Czuba M. Kempa K. Kimsa E. (2012). Acute metabolic responses to a 24-h ultra-marathon race in male amateur runners. *Eur. J. Appl. Physiol.* 112 1679–1688. 10.1007/s00421-011-2135-5 21879351 PMC3324692

[B57] Wu H. J. Chen K. T. Shee B. W. Chang H. C. Huang Y. J. Yang R. S. (2004). Effects of 24 h ultra-marathon on biochemical and hematological parameters. *World J. Gastroenterol.* 10 2711–2714. 10.3748/wjg.v10.i18.2711 15309724 PMC4572198

